# Lifestyle management of polycystic ovary syndrome: a single-center study in Bosnia and Herzegovina

**DOI:** 10.3934/publichealth.2020041

**Published:** 2020-07-08

**Authors:** Jasmina Djedjibegovic, Aleksandra Marjanovic, Ilhana Kobilica, Amila Turalic, Aida Lugusic, Miroslav Sober

**Affiliations:** Faculty of Pharmacy, University of Sarajevo, Sarajevo, Bosnia and Herzegovina

**Keywords:** lifestyle management, family medicine, internal medicine, PCOS, women's health

## Abstract

**Background:**

Polycystic ovary syndrome (PCOS) is a complex endocrinopathy affecting up to 20% of pre-menopausal women. The most recent international guidelines set lifestyle management as the cornerstone of the PCOS treatment. Still, there is a paucity of data on the implementation of lifestyle management in clinical practice. This cross-sectional study aimed to explore physicians-reported practices in PCOS lifestyle management in the Sarajevo Canton, Bosnia and Herzegovina (BiH). The profession of dietetics is not legally recognized in BiH. Nutritional interventions in health promotion and disease treatment are provided by medical professionals.

**Methods:**

Data were collected by a paper-based questionnaire distributed during March-May 2018 in the Public Institution Health Centre of Sarajevo Canton.

**Results:**

Forty-six physicians (response rate of 80.7%) completed the questionnaire. An initial treatment plan based solely on lifestyle measures (diet + physical activity), as recommended by current guidelines was reported by 34.8% of physicians. Although dietary interventions were rated as highly relevant in PCOS management by the vast majority of physicians, only one-half reported recording patients' adherence and 45.7% of physicians were unsure of the effectiveness of the lifestyle interventions in their patients.

**Conclusion:**

PCOS lifestyle management in the study setting is sub-optimal. Additional education on effective PCOS lifestyle management strategies would be beneficial, especially for physicians with less than 15 years in practice. Possible obstacles to better physicians' engagement in PCOS lifestyle management should be further investigated.

## 1. Introduction

Polycystic ovary syndrome (PCOS) is the most common endocrine disorder in pre-menopausal women reported to affect up to 20% of this population group [1]. The most widely used Rotterdam diagnostic criteria for PCOS is the presence of at least two of the following three features: oligo/anovulation, hyperandrogenism, and polycystic ovaries [2]. Women with PCOS usually suffer from infertility, and metabolic abnormalities (impaired glucose tolerance/insulin resistance, obesity, dyslipidemia) put them at high risk for type 2 diabetes and cardiovascular diseases. Insulin resistance was reported to be present in as much as 75% of non-obese and up to 95% of obese women with PCOS [3]. Insulin resistance makes patients more prone to weight gain, and obesity further exacerbates insulin resistance and other biochemical and clinical characteristics of PCOS. Conversely, even a modest weight loss of 5–10% was shown to significantly improve both health and quality of life in women with PCOS [3]. These findings, together with the fact that the etiology of PCOS remains unclear and the treatment is symptomatic, resulted in recognition of lifestyle interventions as the basis for PCOS management. Current international guidelines recommend lifestyle management as the first-line treatment that should be provided to all patients irrespective of the PCOS phenotype [4],[5]. It should be adopted in the integrated model of care for PCOS, which is provided by different medical professionals (general practitioners, nurses, gynecologists, endocrinologists) [6]. However, there is a paucity of data regarding the implementation of lifestyle management in the overall treatment of PCOS. One recent survey among endocrinologists and gynecologists in Europe showed that metformin was the first (33%), and lifestyle modification was the second treatment choice, reported by only 25% of respondents [7]. Primary health care (PHC) physicians have a specific role and position within a health system and should be able to effectively manage lifestyle interventions both in the general population and in specific groups like PCOS patients. Still, the research on physicians' performance in this field is scarce, evidence regarding their effectiveness is lacking, and various barriers have been reported in a few studies published to date [8],[9].

Bosnia and Herzegovina (BiH) is an upper-middle-income country in the Western Balkans, Southeastern Europe. The healthcare system is based on the principle of solidarity with health insurance coverage of more than 85% of the population. The number of physicians per 1000 people is 1.89 (data from 2013) indicating high physicians' workload. The primary health care in BiH is still in the reform towards a new model of PHC centered on family medicine. The main public health care institution in Sarajevo Canton and the largest primary health care institution in BiH, the Public Institution Health Centre of Sarajevo Canton, has established the new Family medicine department, but it also includes several consultative-specialist services that are available to patients only with a family physician's referral. The institution has 9 health centers and provides health care to a population of about 490,000. The dieticians are not available within a public health system as this is not a legally recognized profession in BiH. Lifestyle interventions in health promotion and disease treatment are within the scope of practice of various medical professionals. However, the physicians' practice regarding PCOS lifestyle management was not previously investigated.

This study aimed to gain insight into physicians' practices in PCOS management within the Public Institution Health Centre of Sarajevo Canton, Bosnia and Herzegovina. Specifically, we wanted to get answers to the following questions:

(1) How often physicians opt for non-pharmacological lifestyle modifications as the first-line treatment for PCOS?

(2) Is this practice associated with physicians' or patients' characteristics?

(3) What dietary factors physicians consider essential and implement in PCOS management?

## 2. Methods

### 2.1. Study design and setting

This cross-sectional study was conducted in five of the nine main health centers of the Public Institution Health Centre of Sarajevo Canton (HCSC) in March-May 2018. The target population comprised of public health physicians ordinarily involved in the treatment of patients with PCOS. Therefore, the questionnaires were distributed within the relevant departments: general/family medicine, schoolchildren health care, and internal medicine. The total number of physicians working in the selected departments was 57. Given the small population size, we used the total population sampling approach and a paper-based mode of delivery. While paper-based surveys are generally more costly and time-consuming in comparison to web-based surveys, the response rate is usually higher in the former. Some studies also report on a higher preference of paper surveys over web surveys among physicians [10], a lower response rate to web surveys among specialists than the other types of doctors [11], and even different response rates among various medical specialties [12]. Our previous experience also showed a somewhat higher response rate and higher completeness of data when using a paper-based in-class survey in comparison to a web-based in-class survey even in the younger population in BiH (university students). Therefore, we decided to use the paper-based questionnaires in this study.

The questionnaires were distributed to the physicians and collected back either through the head of the discipline or directly from the researcher. In the latter case, the researcher who distributed and collected the questionnaires (IK) was responsible for keeping the participant's identity confidential (i.e. this information was not shared with any other person, including the members of the research team).

The Institute for Women Health and Maternity in Sarajevo Canton, which is a separate public health institution, was also invited to participate but decided not to take part in the survey owing to physicians' work overload.

The criteria for exclusion were: having no previous experience in PCOS diagnosis or treatment, or incomplete questionnaire (>5 missing answers).

### 2.2. Survey questionnaire

The questionnaire contained 18 questions, including three questions about physician's characteristics (gender, the field of specialization, and years in practice), six questions about the physician's perception of patients' characteristics and nine questions concerning the physician's practice and beliefs in PCOS management (Supplementary file S1). Participants were asked not to fill the questionnaire if they never had a patient with PCOS.

The main outcome variable was the physicians' initial treatment choice (pharmacotherapy, changes in diet, increase in physical activity, dietary supplements or herbal drugs, or other-specify). These originally reported categories were subsequently collapsed into two groups (with medicines or without medicines) for further statistical analysis.

### 2.3. Statistical analysis

We used descriptive statistics (counts and percentages) for the presentation of both reported and missing data. The association between the main outcome variable (initial treatment choice) and independent variables (physicians-reported patients' characteristics and physicians' characteristics) was examined using the Pearson Chi-Squared test or Fisher's Exact test. P-values <0.05 were considered statistically significant. Data analysis was performed using SPSS 23.0 (IBM Inc., New York, USA). The dietary factors reported as essential in PCOS treatment and the type of dietary measures advised to patients were analyzed qualitatively.

### 2.4. Ethics consideration

Participation in the survey was voluntary and anonymous. The survey was approved by the Ethical Committee of the Public Institution Health Centre of Sarajevo Canton before the distribution of the questionnaire. Written informed consent was signed by all participants.

## 3. Results

### 3.1. Study population

In total, 49 of 57 questionnaires were returned (response rate 86.0%). However, three questionnaires were excluded in line with the predefined exclusion criteria, so the final number of questionnaires available for analysis was 46.

Most of the respondents were women (91.3%), specialists in family medicine (60.9%) with more than 20 years in practice (50.0%) (Table 1). Physicians' work experience ranged from 3.5 to 38 years, with an average of 20.7 years (SD = 10.18 years). The similar structure of health professionals employed in the HCSC was reported by other authors (84.4% women; average physicians work experience 20.2 ± 10.74 years) [13]. Thus, we can be confident that our sample is a good representation of the target population in the HCSC.

Statistically significant (p = 0.038) association was found between the initial treatment choice and physicians' working experience; physicians with ≤15 years of work experience tend to include pharmacotherapy in the initial treatment plan more often than their senior colleagues (Table 1). The physicians' field of specialization or gender was not associated with the initial treatment choice, although the latter should be interpreted with caution due to a very small number of male participants (Table 1).

**Table 1.** Physicians' characteristics and their association with the initial treatment choice.

Characteristic	Variable category	Total		With med.^a^		Without med.^a^		p-value
		n	%	n	%	n	%	
Gender	Male	3	6.5	0	0.0	3	6.8	0.100^b^
	Female	42	91.3	23	52.3	18	40.9	
	Missing	1	2.2					
Medical speciality	Family medicine	28	60.9	14	36.8	13	34.2	1.000^b^
	Internal medicine	11	23.9	6	15.8	5	13.2	
	Missing	7	15.2					
Years of working experience	≤15	18	17.4	13	28.9	5	11.1	0.038^c^
	>15	28	50.0	11	24.4	16	35.6	
	Missing	0	0.0					

Note: ^a^ Discrepancies in the totals are due to missing values (initial treatment choice not reported); ^b^ p-Values were obtained using the Fisher's Exact test; ^c^ p-Value was obtained using the Pearson Chi-Square test.

### 3.2. Physicians-reported patients' characteristics

Since characteristics of patients usually seen by physicians of different specialties may be different, and this can affect physicians' therapeutic approach, we have asked our participants about the characteristics of their PCOS patients. The results are shown in Table 2.

The presence of other comorbidities was reported by 30 (65.2%) physicians. Hypothyreosis was reported by 13 (28.3%), diabetes mellitus by 12 (26.1%), insulin resistance by 8 (17.4%), both hirsutism and arterial hypertension were reported by 4 physicians (8.7%), acne and dyslipidemia were reported by two physicians (4.3%), and other comorbidities (anxiety, impaired glucose tolerance, hyperinsulinemia, metabolic syndrome, and hyperprolactinemia) were each reported by one participant.

The patients' characteristics reported by physicians were not associated with the physicians' initial treatment choice (Table 2).

**Table 2.** Patients' characteristics as reported by physicians and their association with the physicians' initial treatment choice.

Patients' characteristic	Variable category	Total		With med.^a^		Without med.^.a^		p-value
		n	%	n	%	n	%	
Age at diagnosis of PCOS (years)	15–25	39	84.8	19	42.2	20	44.4	0.670^b^
	>25	6	13.0	4	8.9	2	4.4	
	Missing	1	2.2					
Prevalence of overweight or obesity (%)	>75	16	34.8	9	20.0	7	15.6	0.915^b^
	50–75	24	52.2	12	26.7	12	26.7	
	<50	5	10.9	3	6.7	2	4.4	
	Missing	1	2.2					
Presence of comorbidities	No	16	34.8	9	20.0	7	15.6	0.771^c^
	Yes	30	65.2	15	33.3	14	31.1	
	Missing	0	0.0					
Prevalence of fertility problems (%)	>75	10	21.7	7	15.6	2	4.4	0.158^b^
	50–75	12	26.1	8	17.8	4	8.9	
	<50	14	30.5	5	11.1	9	20.0	
	Not sure	10	21.7	4	8.9	6	13.3	
	Missing	0	0.0					
Presence of pregnancy complications (%)	No	7	15.2	4	8.9	2	4.4	0.823^b^
	Yes	9	19.6	5	11.1	4	8.9	
	Not sure	30	65.2	15	33.3	15	33.3	
	Missing	0	0.0					
Preferred type of therapy	Pharmacological	31	67.4	17	37.8	14	31.1	0.900^b^
	Non-pharmacological	9	19.6	5	11.1	4	8.9	
	Not sure	5	10.9	2	4.4	3	6.7	
	Missing	1	2.2					

Note: ^a^ Discrepancies in the totals are due to missing values (initial treatment choice not reported); ^b^ p-Values were obtained using the Fisher's Exact test; ^c^ p-Value was obtained using the Pearson Chi-Square test. With med.—initial treatment includes medicines; Without med.—initial treatment does not include medicines.

### 3.3. Physicians-reported PCOS management practices

**Figure 1.**
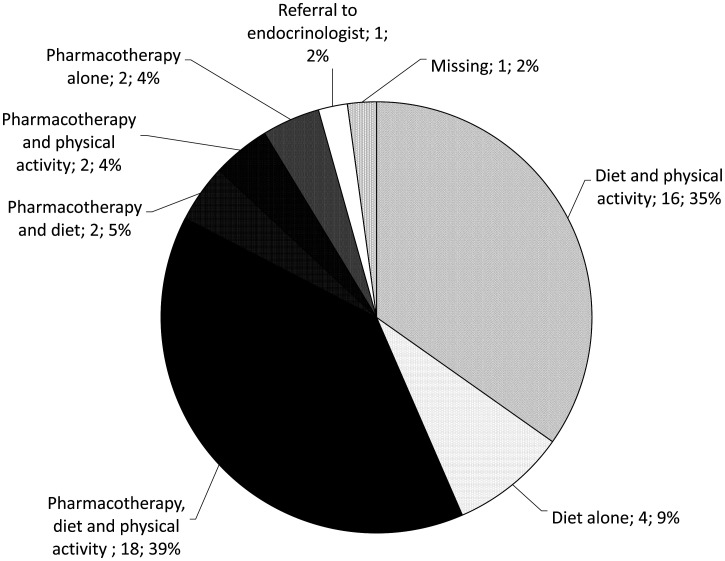
The frequencies (n; %) of different initial PCOS treatments reported by physicians.

**Figure 2.**
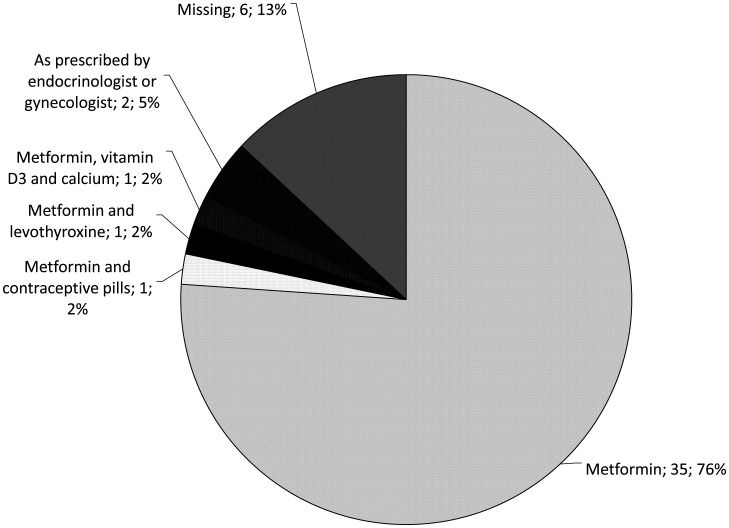
Most often prescribed medicines in PCOS management.

The initial treatment choice based solely on lifestyle modification and including both diet and physical activity was reported by 16 (34.8%) of physicians, while the rest of them reported to include medication in the initial treatment plan (Figure 1). The most often prescribed medicine was metformin (n = 38; 82.6%), either alone (n = 35; 76.1%) or with other medicines (n = 3; 6.5%) (Figure 2).

Physicians' practices and beliefs concerning PCOS management are further presented in Table 3. The vast majority of physicians (39/46; 84.8%) rated dietary measures as highly important (4–5 points on a 1–5 Likert scale) in PCOS management. Interestingly, this opinion was not associated with the physician's initial treatment choice for their patients with PCOS (Table 3).

**Table 3.** The physicians' practice and beliefs and their association with the physicians' initial treatment choice.

Question	Answers	Total		With med.^a^		Without med.^a^		p-value
		n	%	n	%	n	%	
How often do you diagnose and/or treat PCOS?	Often	14	30.4	6	13.3	8	17.8	0.546^b^
	Rarely	28	60.9	15	33.3	12	26.7	
	Very rarely	4	8.7	3	6.7	1	2.2	
	Missing	0	0.0					
In your opinion, how important is a diet in PCOS management?	2	3	6.5	1	2.2	1	2.2	0.654^b^
	3	4	8.7	2	4.4	2	4.4	
	4	13	28.3	9	20.0	4	8.9	
	5 (extremely important)	26	56.5	12	26.7	14	31.1	
	Missing	0	0.0					
Do you typically advise patients to use dietary supplements or herbal medicines?	Yes	8	17.4	5	11.4	3	6.8	0.701^b^
	No	37	80.4	18	40.9	18	40.9	
	Missing	1	2.2					
Do you record patients' adherence to the advised dietary and lifestyle changes?	Yes	23	50.0	11	25.0	12	27.3	0.349^c^
	No	22	47.8	13	29.5	8	18.2	
	Missing	1	2.2					
Does the implementation of lifestyle modifications typically improve therapeutic outcomes in your patients?	Yes	22	47.8	9	20.0	13	28.9	0.103^b^
	No	3	6.5	1	2.2	2	4.4	
	Not sure	21	45.7	14	31.1	6	13.3	
	Missing	0	0.0					

Note: ^a^ Discrepancies in the totals are due to missing values (initial treatment choice not reported); ^b^ p-Values were obtained using the Fisher's Exact test; ^c^ p-Value was obtained using the Pearson Chi-Square test. With med.—initial treatment includes medicines; Without med.—initial treatment does not include medicines.

In total, about one-half of participants reported recording patients' adherence to the prescribed lifestyle measures. This practice was not significantly associated with the physicians' initial treatment choice (Table 3). Similarly, about one-half of physicians reported that they are not sure whether lifestyle measures improve therapeutic response in their patients. The physicians' initial treatment choice was not associated with the perceived effectiveness of lifestyle measures in their patients with PCOS.

When asked what dietary and lifestyle measures do they advise to their PCOS patients, the physicians mostly responded with general terms: healthy diet (16/46; 34.8%), hygienic-dietary regimen (12/46; 26.1%), physical activity (29/46; 63.0%), and lifestyle changes (8/46; 17.4%). The weight control/reduction was specifically mentioned by 6 (13.0%), and smoking cessation by 3 (6.5%) physicians. The physical activity was specified in terms of duration (at least 30 minutes per day or equivalent) and/or intensity (moderate to vigorous) by 5 physicians (10.9%).

Among the key dietary factors in PCOS management, decreased intake of carbohydrates (including glucose) was listed by 32.6% (15/46), decreased intake of fat (including trans-fats and animal fat) was listed by 8.7% (4/46), while decreased intake of both carbohydrates and fats was listed by 30.4% (14/46) of physicians in our study (Table 4). Specific dietary patterns were also reported in individual cases (food combining diet, blood type diet, diabetes diet, energy-restricted diet, Mediterranean diet).

**Table 4.** The most important dietary factors in PCOS treatment as reported by physicians (n = 46).

Dietary pattern	Frequency		Foods	Frequency		Nutrients	Frequency	
	n	%		n	%		n	%
Regular daily meals	4	8.7	↑ fruits	9	19.6	↓ carbohydrates	28	60.9
Energy-restricted diet	3	6.5	↑ vegetables	8	17.4	↓ glucose	1	2.2
Diabetes diet	1	2.2	↑ wholegrain cereals/foods	3	6.5	↑ dietary fibers	2	4.3
Mediterranean diet	1	2.2	↑ nuts	3	6.5	↓ fats	15	32.6
Food combining diet	1	2.2	↑ white meat (chicken, turkey)	2	4.3	↓ salt	3	6.5
Blood type diet	1	2.2	↑ fish	2	4.3	↓ fats of animal origin	2	4.3
			↑ legumes	1	2.2	↓ trans-fats	1	2.2
			↓ sugar-sweetened beverages	5	10.9	Balance of vitamins and minerals	1	2.2
			↓ sweets	3	6.5	Vitamin D supplementation	1	2.2
			↓ white flour	2	4.3			
			↓ high glycemic index food	1	2.2			
			↓ coffee	2	4.3			
			↓ alcoholic drinks	1	2.2			
			↓ fast food	1	2.2			
			↓ processed food	1	2.2			
			↓ pasta	1	2.2			
			↓ fried food	1	2.2			

Note: ↑—increased consumption; ↓—decreased consumption.

**Table 5.** Dietary supplements advised to patients with PCOS.

Participant ID	Supplements
A3	Evening primrose oil, noni juice, complex herbal formula (*Alchemillae folium, Millefolii herba, Petroselini fructus, Calendulae flos, Geranii robertiani herba, Polygoni herba*)
A17	Vitamin D3
A24	Maca root preparation
A34	Vitamin D3, calcium, herbal tea (not specified)
A44	Vitamin E, vitamin D

Eight physicians (17.4%) reported to usually advise the use of dietary supplements or herbal drugs to their patients with PCOS, including vitamins D and E, calcium, Maca root preparation, noni juice, Evening primrose oil, and one complex herbal supplement (Table 5).

## 4. Discussion

To the best of our knowledge, this type of study on physicians' practices concerning lifestyle management of PCOS was the first in Bosnia and Herzegovina. This type of study is also rare in the literature irrespective of the study area.

The presence of diabetes mellitus and risk factors for diabetes mellitus type 2 (insulin resistance, impaired glucose tolerance, hyperinsulinemia, and metabolic syndrome) in PCOS patients were reported by 12/46 (26.1%) and 11/46 (23.9%) of participants in our study, respectively. In the North Europe study, conducted among 382 doctors (75% obstetrician-gynecologists, 25% endocrinologists; 79% women) from 6 countries, the increased risk for diabetes mellitus type 2 and insulin resistance in PCOS patients were reported by 95% and 97% of participants, respectively [14]. Although this may seem like a huge discrepancy in comparison to our data, it must be noted that prevalence of insulin resistance in PCOS patients reported in the literature range from 44% to 70% [15], and even higher prevalence (83.3% in lean and 93.1% in overweight women) was reported among the PCOS patients presented with infertility [16]. Besides certain patients' characteristics (ethnicity, genetic factors, body weight, and visceral adiposity), the detection of IR among women with PCOS was shown to be highly method-dependent with a higher detection rate obtained with the insulin resistance (Belfiore) index than with HOMA-IR [17].

The prevalence of hypothyreosis in women with PCOS was reported by 13 (28.3%) physicians in our study. There is emerging evidence that thyroid disorders are more common in women with PCOS than in the normal population, although this phenomenon and its clinical relevance are yet to be explained [18].

Fertility problems and pregnancy complications were reported by a lower percentage of physicians in our study (78.3% and 19.6%, respectively) than in the North Europe study (96% and 53%, respectively) [14]. This difference could be explained by the fact that participants in the North Europe study were mainly obstetrician-gynecologists. However, 21.7% of our study participants were not aware of the prevalence of fertility problems, and 65.2% were not aware of the prevalence of pregnancy complications in their patients with PCOS (Table 2). This finding possibly points out a fragmentation by specialty in the healthcare system and a lack of communication between the health professionals, which has been shown to result in delays to diagnosis and treatment and exacerbate dissatisfaction in patients with PCOS [19],[20].

The anxiety among women with PCOS was reported by 24% of participants in the North Europe study [14], while it was mentioned by only one physician (2.2%) in our study. The results suggest that physicians are not fully aware of psychosocial comorbidities in women with PCOS. Indeed, the prevalence of depression and anxiety was reported to be up to three times higher and with more severe symptoms in patients with PCOS than in women without PCOS [21]–[25]. These symptoms can further affect a patient's motivation to follow the treatment plan and have been linked to adverse outcomes [26]. Although the research on effects of lifestyle modifications on mental health in PCOS is still modest, there is some evidence that regular exercising is associated with fewer symptoms of anxiety and depression [27], and patients who reported to have at least 150 minutes of moderate exercise each week were less likely to be depressed [28].

Of all respondents, 52.2% (24/46) reported medical therapy as a part of the initial treatment, which is not recommended by the current guidelines. Physicians with no more than 15 years in practice reported to include pharmacotherapy in the initial treatment more often than their colleagues with longer work experience (p = 0.038). In the North Europe study, the physicians aged <35 years were 2.2 times more likely to recommend lifestyle management for patients with PCOS for fertility concerns than their older colleagues [14].

According to the most recent international PCOS management guidelines, lifestyle interventions (preferably all three of the following: diet, exercise, and behavioral management strategies) should be recommended in all PCOS patients, and especially those with excess weight [4]. In line with those recommendations, the majority of participants (88.0%) in our study reported some dietary measures as a part of their initial PCOS treatment strategy (Figure 1). Still, the advice on weight reduction/management was explicitly mentioned by only 6 physicians (13.0%), and even a lower number (n = 3; 6.5%) specified energy-restricted diet as one of the key dietary factors in PCOS management (Table 4). This was somewhat surprising since even a modest weight reduction of 5–10% can improve many (clinical, hormonal, and metabolic) disorders in PCOS, and 87% of physicians reported a high prevalence (≥50%) of obesity among their patients. In the North Europe study, “increased tendency for weight gain” in women with PCOS was reported by 84% of the participants, and “difficulties losing weight” by 79% of participants [14]. Thus, the weight management problem in PCOS is well recognized by physicians. Lifestyle modifications were the most common treatment for fertility concerns in PCOS prescribed by 56% of participants, and the second most common treatment for non-fertility-related PCOS concerns (after the oral contraceptives) prescribed by 66% participants in the North Europe study. Nevertheless, remarkable differences between countries were noted. In Norway, lifestyle modification was reported as the most common PCOS treatment prescribed more often than any medication, while in Estonia medications were prescribed more often (clomiphene citrate with metformin for fertility-related concerns; oral contraceptives and metformin for non-fertility-related concerns) [14]. Another important point that must be considered is patient satisfaction with the service provided by health professionals. In this context, it is worth noting that one recent study conducted among 1385 women from 32 countries worldwide found that over half of women reported receiving lifestyle management information at PCOS diagnosis, but only 12% were satisfied with it [29].

Although the European Society for Human Reproduction and Embryology (ESHRE) and American Society for Reproductive Medicine (ASRM) endorsed lifestyle counseling in PCOS management, it is not as yet a regular clinical practice, and medication therapy is still preferred first-line treatment in some countries. The most often used medicines in the last few decades were metformin and clomiphene citrate [14],[30]. Metformin was also reported as the most often prescribed medication in our survey (Figure 2). Metformin improves not only insulin resistance but also anovulatory infertility [31], and both of these problems were reported as patients' characteristics in our study.

The majority of physicians (78.3%) reported to include physical activity in the initial PCOS treatment plan (Figure 1). The advice on physical activity was specified in terms of duration and/or intensity by only 5 (10.9%) physicians. Both diet and exercise as a sole initial PCOS treatment plan, as recommended in the guidelines, was reported by only 16 (34.8%) physicians. Furthermore, none of the participants stated any of the behavioral strategies as a part of the PCOS treatment plan. This may be one possible explanation for the high percentage of physicians (67.4%) who believe that patients accept pharmacological treatment better than the lifestyle modifications. While lifestyle advice could indeed be quite simple, its implementation and maintenance in everyday life are far more demanding and can be improved by behavioral therapy [3],[32]–[34]. Thus, if the behavioral component is not a part of the treatment plan one could expect a lower adherence among patients, especially in the long term.

Current guidelines for PCOS management state that there is no enough evidence that any specific energy equivalent diet type is significantly better than another. The most important dietary intervention is energy restriction/control, which was explicitly mentioned as the key dietary factor in PCOS management by only 3 (6.5%) physicians in our study. Listing the key dietary factors in PCOS management physicians mainly focused on macronutrients; 32.6% favored decreased intake of carbohydrates, 8.7% favored decreased intake of fat, while decreased intake of both carbohydrates and fats was listed by 30.4% of physicians. Although the available evidence is not straightforward, most of the published data were in favor of somewhat lower carbohydrates intake and low glycemic index, i.e. low glycemic load diet [35],[36], while fat limitation to <30% of daily energy could be a reasonable strategy for energy restriction (to reduce body weight in obese patients) and could also be advised to patients whose regular fat intake is too high [37]. The Food combining diet and Blood type diet, each noted as a key dietary factor in PCOS management by one of the participants in our study are not evidence-based dietary interventions. This finding indicates that some physicians may be using unreliable sources of information.

Although 84.8% of physicians rated dietary interventions as highly important in PCOS management (4–5 points on a 1–5 Likert scale), still 47.8% of them stated that they don't record patients' adherence to lifestyle modifications. Furthermore, 47.8% (22/46) of physicians in our study reported on typically positive effects of the lifestyle modifications in their patients with PCOS (Table 3), while another 45.7% (21/46) were unsure of the effects. A non-standardized delivery of lifestyle management and lack of enforcement and follow up of interventions were also identified as systemic barriers to lifestyle management implementation in PCOS in Singapore [38]. Similar findings were also reported by other authors; Arasu et al. [9] reported on general practitioners' acknowledgment of the importance of weight and lifestyle management in PCOS, but their practices were influenced by various barriers (time constraints, lack of financial reimbursement, weight management being professionally unrewarding, perceived lack of patient motivation, costs of accessing allied health professionals and their unavailability in certain locations). While we did not examine such factors in our study, at least some of the above-mentioned barriers could easily be recognized as present in our health care system as well and were indeed reported in the recent study investigating factors influencing the implementation of family medicine in Bosnia and Herzegovina [39]. Three participants reported advising vitamin D supplements. Recent systematic review and meta-analysis of randomized controlled trials suggest a possible benefit of vitamin D supplementation for follicular development and regulation of the menstrual cycle in PCOS patients [40]. In a randomized control trial involving 90 insulin-resistant PCOS patients, high-dose vitamin D (4000 IU) had beneficial effects on total testosterone, sex hormone-binding globulin, free androgen index, serum hs-CRP, and plasma total antioxidant capacity levels compared with low-dose vitamin D (1000 IU) and placebo groups [41],[42]. Co-supplementation with calcium and vitamin D (Ca 1000 mg/day, vitamin D 6000 IU/day during 8 weeks) was shown to significantly improve menstrual cycle regularity when added to metformin (1500 mg/day) in the randomized placebo-controlled clinical trial in 40 vitamin D-deficient PCOS patients [43]. Similar results were reported in a few more randomized clinical trials, although with higher vitamin D dose [44],[45]. Vitamin E was shown to have beneficial effects on the metabolic and clinical signs of PCOS, alone or in combination with coenzyme Q10 and omega-3 fatty acids [46]–[49]. Two physicians reported advising the use of herbal supplements (Table 5). Even though Evening primrose (Oenothera biennis L) oil, noni (Morinda citrifolia L) juice, and Maca (Lepidium meyenii Walp) are all described in traditional medicine, we were not able to find clinical trials which support their use specifically in PCOS treatment. The complex herbal drops mentioned by one physician are marketed as a dietary supplement and advertised for PCOS treatment, but the scientific evidence for the claimed effect is not publicly available.

In summary, this study suggests that physicians are well aware of the guidelines on a healthy diet and lifestyle in general, but they seem to be less well equipped for adequate lifestyle counseling in women with PCOS. The reported practices and beliefs, and advice to patients are not consistent among physicians, which is probably due to a lack of structured lifestyle counseling program. Physicians' engagement in this type of treatment is also quite low, as evidenced by the high percentage of physicians who did not record patients' adherence to the prescribed lifestyle treatment and were unsure of the effects of such treatment in their PCOS patients.

Since PCOS is a complex disease and a serious global health concern, patient care needs to be multidisciplinary and well-coordinated. In some countries (e.g. Australia), such model of care was already introduced as a specialized PCOS clinic. Unfortunately, many countries are still not doing very well in diagnosing PCOS and adequately supporting women in learning how to make lifestyle changes.

### 4.1. Strengths and limitations

The study setting and high response rate (80.7%) ensure that collected data give a reliable description of PCOS lifestyle management practice among family physicians and internal medicine specialists in the Public Institution Health Centre of Sarajevo Canton. However, gynecologists did not participate in the study, thus our findings are not a complete overview of the PCOS lifestyle management within the public health care in the study area. Furthermore, the findings may not represent physicians' practices in the entire country and could be of limited relevance in different settings. Another important point is that we only included a limited number of variables, while many other factors could influence the physicians' practice. Still, the presented results are a good starting point for further research. The authors also acknowledge that the number of participants in subgroups was occasionally small, thus some between-group differences were possibly not detected simply due to the low statistical power of the study. Despite these limitations, the study adds to a very limited data on this important issue.

## 5. Conclusion

Based on the study results, the answers to the research questions are the following:

(1) The first-line PCOS treatment in accordance with the current guidelines (diet and physical activity) was reported by 34.8% of physicians.

(2) Physicians with >15 years in practice include medicines in the initial PCOS treatment plan less often than their younger colleagues. Physicians' medical speciality or patients' characteristics were not associated with the reported first-line treatment. However, the findings of the present study suggest that the family physicians and internal medicine specialists in the Public Institution Health Centre of Sarajevo Canton are insufficiently engaged in PCOS lifestyle management.

(3) Physicians are very well informed about a healthy lifestyle in general, but the most important points specifically in PCOS lifestyle management were only rarely reported. Some of the reported dietary interventions are not based on adequate scientific evidence and best practices.

The results of the present study also suggest that psychosocial comorbidities in women with PCOS seem to be largely overlooked in the study population, supporting similar findings from other European countries. Thus, the additional education specifically on lifestyle counseling in women with PCOS could be beneficial, particularly for physicians with less working experience. Possible other obstacles to better physicians' engagement in PCOS lifestyle management should be further investigated.
