# Supplementary material for: Lifestyle management of polycystic ovary syndrome: a single-center study in Bosnia and Herzegovina
Source: AIMS Public Health. 2020 Jul 8;7(3):504–20. doi: 10.3934/publichealth.2020041 (PMC7505790; doi:10.3934/publichealth.2020041)
Supplement: Supplementary file 1 [file publichealth-07-03-041-s001.pdf]

AIMS Public Health

AIMS Public Health, 7(3): 504–520.
DOI: 10.3934/publichealth.2020041
Received: 17 May 2020
Accepted: 07 July 2020
Published: 08 July 2020

http://www.aimspress.com/journal/aimsph

Research article

Lifestyle management of polycystic ovary syndrome: a single-center study in Bosnia and Herzegovina

Jasmina Djedjibegovic*, Aleksandra Marjanovic, Ilhana Kobilica, Amila Turalic, Aida Lugusic and Miroslav Sober

Faculty of Pharmacy, University of Sarajevo, Sarajevo, Bosnia and Herzegovina

* **Correspondence:** Email: djedjibegovicj@gmail.com; Tel: +38733586178.

Supplementary

File S1. The study questionnaire (English translation).

This questionnaire is intended for medical doctors involved in the treatment of patients with polycystic ovary syndrome. It is designed to collect the data for a study that aims to assess the counseling practice regarding non-pharmacological adjuvant treatment (diet, lifestyle, dietary supplements) in the polycystic ovary syndrome. The study is conducted as a part of a work on a master thesis "Nutrition support in polycystic ovary syndrome" to be defended at the Faculty of Pharmacy – University of Sarajevo.
Thank you for your time and cooperation.

Questionnaire

Gender: M F

Your field of specialization/subspecilization: ______________________________

Your working experience (years): ______________________________

1. How often do you diagnose and/or treat PCOS in your practice?

a) Often
b) Rarely
c) Very rarely
d) I never had a patient with PCOS in my practice.

If your answer is d) please don’t fill the questionnaire.

2. At what age are your patients usually diagnosed with PCOS?

a) Before the age of 15 years
b) At 15-25 years of age
c) After the age of 25 years

3. How often are your patients with PCOS overweight or obese at diagnosis?

a) In >75% of cases
b) In 50-75% of cases
c) In 25-49% of cases
d) In <25% of cases

4. What is your initial treatment choice for the new diagnosed PCOS patients? (you can mark multiple answers)

a) Pharmacotherapy
b) Dietary changes
c) Increased physical activity
d) Dietary supplements and herbal therapy
e) Other (please specify):
____

5. What kind of treatment is better accepted by your patients?

a) Pharmacological
b) Non-pharmacological (lifestyle modifications)
c) Not sure

6. How often your patients with PCOS have reproductive problems?

a) In >75% of cases
b) In 50-75% of cases
c) In 25-49% of cases
d) In <25% of cases
e) Not sure

7. Do your patients with PCOS usually suffer from other comorbidities?

a) Yes
b) No

If your answer is “Yes”, please list the comorbidities:

____

8. Do your patients with PCOS usually have complications in pregnancy?

a) Yes
b) No
c) Not sure

9. What medicines do you prescribe most often to your patients with PCOS?

____

10. If you advise non-pharmacological treatment to your patients with PCOS, please list the measures you advise most often:

____

11. In your opinion, how important is a diet for success in PCOS treatment? (1—not important at all, 5—extremely important)

1 2 3 4 5

12. What dietary factors do you consider crucial in PCOS treatment? Please list:

____

13. Do you typically advise the use of dietary and/or herbal supplements to your patients with PCOS?

a) Yes
b) No

If your answer is "Yes", please specify:______________________________________

14. Do you record patients' adherence to the advised dietary and lifestyle changes?

a) Yes
b) No

15. Does the implementation of non-pharmacological treatment measures typically improve the therapeutic outcomes in your patients with PCOS?

a) Yes
b) No
c) Not sure
